# Regulatory B Cells Expressing Granzyme B from Tolerant Renal Transplant Patients: Highly Differentiated B Cells with a Unique Pathway with a Specific Regulatory Profile and Strong Interactions with Immune System Cells

**DOI:** 10.3390/cells13151287

**Published:** 2024-07-31

**Authors:** Nicolas Sailliet, Amandine Dupuy, François Brinas, Karine Renaudin, Luc Colas, Clarisse Kerleau, Thi-Van-Ha Nguyen, Cynthia Fourgeux, Jérémie Poschmann, Clément Gosset, Magali Giral, Nicolas Degauque, Hoa Le Mai, Richard Danger, Sophie Brouard

**Affiliations:** 1 CHU Nantes, Nantes Université, INSERM, Center for Research in Transplantation and Translational Immunology (CR2TI), UMR 1064, ITUN, 44000 Nantes, France; nicolas.sailliet@univ-nantes.fr (N.S.); francois.brinas@univ-nantes.fr (F.B.); karine.renaudin@chu-nantes.fr (K.R.); luc.colas@univ-nantes.fr (L.C.); clarisse.kerleau@chu-nantes.fr (C.K.); elisabeth.nguyen@univ-nantes.fr (T.-V.-H.N.); cynthia.fourgeux@univ-nantes.fr (C.F.); jeremie.poschmann@univ-nantes.fr (J.P.); magali.giral@chu-nantes.fr (M.G.); nicolas.degauque@univ-nantes.fr (N.D.); le.hoa.mai@univ-nantes.fr (H.L.M.); richard.danger@univ-nantes.fr (R.D.); 2 CHU Nantes, Service d’Anatomie et Cytologie Pathologiques, 44000 Nantes, France; 3 Service de Néphrologie et Transplantation rénale—CHU Pasteur2, 06000 Nice, France; gosset.c@chu-nice.fr; 4 Centre d’Investigation Clinique en Biothérapie, Centre de Ressources Biologiques (CRB), CHU Nantes, 44000 Nantes, France; 5 LabEx IGO “Immunotherapy, Graft, Oncology”, Nantes Université, 44000 Nantes, France

**Keywords:** regulation, B cells, transplantation, kidney, lymphocyte, tolerance

## Abstract

The aim of our study was to determine whether granzyme B-expressing regulatory B cells (GZMB^+^ B cells) are enriched in the blood of transplant patients with renal graft tolerance. To achieve this goal, we analysed two single-cell RNA sequencing (scRNAseq) datasets: (1) peripheral blood mononuclear cells (PBMCs), including GZMB^+^ B cells from renal transplant patients, i.e., patients with stable graft function on conventional immunosuppressive treatment (STA, *n* = 3), drug-free tolerant patients (TOL, *n* = 3), and patients with antibody-mediated rejection (ABMR, *n* = 3), and (2) ex-vivo-induced GZMB^+^ B cells from these groups. In the patient PBMCs, we first showed that natural GZMB^+^ B cells were enriched in genes specific to Natural Killer (NK) cells (such as *NKG7* and *KLRD1)* and regulatory B cells (such as *GZMB*, *IL10*, and *CCL4*). We performed a pseudotemporal trajectory analysis of natural GZMB^+^ B cells and showed that they were highly differentiated B cells with a trajectory that is very different from that of conventional memory B cells and linked to the transcription factor KLF13. By specifically analysing GZMB^+^ natural B cells in TOLs, we found that these cells had a very specific transcriptomic profile associated with a reduction in the expression of HLA molecules, apoptosis, and the inflammatory response (in general) in the blood and that this signature was conserved after ex vivo induction, with the induction of genes associated with migration processes, such as *CCR7*, *CCL3*, or *CCL4*. An analysis of receptor/ligand interactions between these GZMB^+/−^ natural B cells and all of the immune cells present in PBMCs also demonstrated that GZMB^+^ B cells were the B cells that carried the most ligands and had the most interactions with other immune cells, particularly in tolerant patients. Finally, we showed that these GZMB^+^ B cells were able to infiltrate the graft under inflammatory conditions, thus suggesting that they can act in locations where immune events occur.

## 1. Introduction

Sustaining solid organ transplantation in the absence of immunosuppression (IS) represents the “Holy Grail of Transplantation”, which is prompted by the description of rare cases of spontaneous operational tolerance (SOT) [1,2]. Although this process remains rare, several groups have attempted to decipher the associated immune mechanisms. In particular, based on an IS withdrawal clinical trial in liver transplantation, we now know that the time after transplantation and the transient infiltration of regulatory T cells are associated with the success of tolerance [3,4]. These results suggest that the immune system and/or the graft environment may evolve towards a pro-tolerogenic profile under immunosuppressive treatment and persist after the cessation of treatment. This scenario was clearly demonstrated in a mouse model of tolerance, with a posttransplant shift from a graft environment rich in T cells to one rich in B cells expressing regulatory markers that contribute to the pro-tolerogenic profile of the graft [5].

Regulatory B cells (Bregs) were identified five decades ago and are increasingly being studied in transplantation; in particular, B cell depletion strategies have been shown to be ineffective in preventing antibody-mediated rejection (ABMR) and may even worsen its progression in humans [6,7,8]. However, the characterisation of Bregs has been complicated by the diversity of B cell subsets with such regulatory properties [9,10]; moreover, until recently, this characterisation has been based mainly on their functions, such as IL-10^+^ [11,12,13,14], IL-35^+^ [15], TGF-β^+^ [16], PD-L1^+^ [17], or GZMB^+^ B cells [18,19,20,21,22] with multiple phenotypes, as well as transitional B cells [23,24,25,26], plasmablasts [27], CD5^+^ B cells [28], CD9^+^ B cells [29], or CD27^−^IgD^−^ B cells [30].

In this study, we focused on Bregs expressing the granzyme B molecule (GZMB^+^ B cells), which we found to be increased in frequency and number in the blood of kidney-transplant-tolerant patients [22]. These GZMB^+^ B cells are also present in the blood of healthy volunteers, wherein they play a role in immune homeostasis [22,31]. GZMB^+^ B cells exhibit different phenotypes, including CD5^+^, IgD^−^CD27^−^, and CD38^+^CD1d^+^IgM^+^CD147^+^ phenotypes, with different levels of differentiation and different origins [9,22,27,28,30,32]. Due to their rarity (approximately 1% of peripheral B cells in healthy volunteers), protocols have been developed to induce them ex vivo [21,32,33,34]. We have shown that stimulation with BCR, CD40L, CpG ODN, IL-21, and IL-2 yields more than 95% of ex-vivo-induced GZMB^+^ B cells after 3 days in culture, starting from total B cells. These induced cells retain their suppressive properties [19].

The mechanisms of GZMB^+^ B cells are not completely understood. We have shown that these cells inhibit the proliferation of effector T cells (CD4^+^/CD8^+^) via mechanisms that are dependent on contact between GZMB^+^ B cells and their target and partially dependent on GZMB [22], in which the transcription in B cells is induced by BATF and CREM after IL-21/JAK stimulation and dependent on lymphotoxin-α (LTα)/CREB [35]. Other studies have shown that GZMB^+^ B cells also induce T-cell apoptosis via TCRζ degradation [21,32,36].

We recently described the transcriptomic profile of these cells in healthy volunteers [35,37] but not in kidney transplant patients; additionally, whether these cells, which are more numerous in tolerant patients, have a specific profile under these conditions of transplantation needs to be explored. Thus, in the present study, we used scRNAseq to analyse the transcriptomes of GZMB^+^ and GZMB^−^ B cells from the blood of transplanted patients in different clinical situations and after ex vivo induction. We analysed (for the first time) the transcriptomic profiles of these cells, their trajectories, and the interactions that these cells develop with other blood cells in transplanted patients. Finally, we demonstrated the presence of these cells in the kidney allograft under inflammatory conditions, such as during rejection processes.

## 2. Materials and Methods

### 2.1. Patients and Samples

We collected blood samples from kidney transplant patients, including (I) spontaneously tolerant patients (TOL, *n* = 4) with no immunosuppression for at least one year and with stable creatinine < 150 mmol/L and proteinuria < 1 g/24, as previously described [38]; (II) patients with stable (STA, *n* = 4) graft function (creatinine < 150 mmol/L and proteinuria < 0.2 g/g creatinine) for at least 3 years under standard immunosuppression (calcineurin inhibitors [CNIs], antimetabolite ± corticosteroids); and (III) patients with histology-proven antibody-mediated rejection (ABMR, *n* = 5). Patient characteristics are provided in Table 1.

### 2.2. Ethics Statements

This study was performed in accordance with the Declaration of Helsinki and approved by the National French Ethics Committee (CPP) N°337/2002, “Characterization of operational tolerance in kidney transplanted recipients without immunosuppressive drugs”. All of the participants who were enrolled in this study signed informed consent forms.

### 2.3. PBMC Isolation

Peripheral blood mononuclear cells (PBMCs) were isolated from whole blood by using Ficoll gradient centrifugation via standard procedures. Following red blood cell lysis, the cells were frozen in foetal calf serum (10% DMSO) and stored at −150 °C until the experiments.

### 2.4. Induction of GZMB^+^ B Cells Ex Vivo

All of the cultures were made in RPMI 1640 (Gibco) containing 10% foetal calf serum, 2 mM L-glutamine, and 100 U/mL penicillin/streptomycin. B cells were enriched by using a Human B cell Isolation Kit II (Miltenyi Biotec, Gladbach, Germany), and separation was performed on an AutoMACS Pro Separator following the manufacturer’s instructions (Miltenyi Biotec). B cells were divided into two fractions: one fraction was left unstimulated, and the second fraction was used to expand GZMB^+^ B cells, as previously described [39]. The following induction cocktail was used: CpG ODN 2006 (1 µg/mL) (InvivoGen, Toulouse, France), soluble rhCD40L (50 ng/mL) (R&D Systems, Minneapolis, MN, USA), rhIL-2 (50 IU/mL) (Proleukin—Novartis), rhIL-21 (10 ng/mL) (R&D Systems), and anti-human IgG/A/M F(ab)’2 (5 µg/mL) (Jackson ImmunoResearch). The cells were cultured in 6-well plates at 10^6^ cells/mL for 72 h at 37 °C and 5% CO_2_. According to this protocol, >95% of induced GZMB^+^ B cells express GZMB and maintain their suppressive activity [39]. These cells are referred to as GZMB^+^ B cells.

### 2.5. Cell Multiplexing and Single-Cell RNA Sequencing

scRNA-seq using the CITE-seq method [40] was essentially performed as described in Abidi et al. [41]. PBMC data were obtained by subsampling the latest timepoint from Laurent et al. [42] corresponding to the sample after rejection for ABMR, long-term stability for STA, and tolerance after IS withdrawal for TOL. For each experiment, the cells were marked with a viability dye (Fixable Viability Dye eFluor 450, 1/1000 in PBS, InvitrogenInvitrogen, Waltham, MA, USA) for 25 min. The living cells were sorted on an ARIA III (BD Biosciences, Franklin Lakes, NJ, USA) and marked with conjugated DNA sequences (HashTag Oligonucleotide, HTO, Chromium Single Cell 3′ Feature Barcode Kit, PN-1000079) that were specific to the donor and the experimental conditions following CITE-seq protocols [40]. The cells were then pooled, and 20,000 total cells were loaded onto a Chromium controller (Chromium Next GEM Single Cell 3′ Kit v3.1; Chromium Next GEM Chip G Single Cell Kit; 10× Genomics). Libraries were prepared and sequenced on a Nova-Seq 6000 (Illumina, San Diego, CA, USA) on the GenoBird platform (Nantes University). Raw reads were analysed by using FastQC for quality control and processed by using the CellRanger pipeline (v3.1.0) with default parameters. The generated FASTQ files were aligned to the reference human genome GRCh38.

### 2.6. scRNAseq Analysis

Analysis was performed by using R (version 4.3.1). The data were analysed by using the Seurat R package [43] (v4.4.0). Cells with fewer than 200 genes and more than 4000 genes or 25% mitochondrial genes were excluded. Azimuth was used to identify cells according to a human PBMC reference [43]. Differential gene expression was calculated by using the MAST R package v1.26.0 [44] with a hurdle model tailored to the scRNAseq data. *p* values were adjusted with FDR, the threshold was set at 0.05, and the log_2_FoldChange threshold was set at 0.2. To retain only genes that were robustly expressed, differential gene expression was calculated only for genes that were expressed in at least 20% of cells in one analysed group. We used Monocle v3 to monitor and compare B cell trajectories [45]. We subsequently used the DecoupleR script in a multivariate linear model to identify the transcription factors that were specifically associated with the different B cell clusters [46]. Gene Ontology (GO) enrichment was performed with the ClusterProfiler package, and term reduction was performed with Revigo [47,48]. We used the Nichenet method (version 2.0.1) to evaluate the expression of receptor–ligand pairs in these data [49]; receptors are present on PBMCs, and ligands are present on natural GZMB^+^ B cells or GZMB^−^ B cells. Nichenet was also used to identify the targets that were differentially expressed as a result of these receptor–ligand interactions. To accomplish this task, Nichenet uses public databases, such as KEGG, ENCODE, Omnipath, and PhosphoSite. A ligand was associated with a cluster if its expression within the cluster was greater than the mean ± standard deviation of its expression across all B cell clusters. Ligands that did not meet this criterion in any cluster were assigned to “general” expression. In addition, only ligands expressed in at least 20% of GZMB^+^ or GZMB^−^ B cells were retained. This analysis was first performed on the natural GZMB^+^ and GZMB^−^ of all patients (TOL, STA, and ABMR) and then on the TOL only (TOL vs. STA and TOL vs. ABMR).

### 2.7. Biopsy Immunofluorescence Staining

To confirm the presence of GZMB^+^ B cells in the allograft, we performed OPAL multiplex immunohistochemistry (IHC) with anti-CD19 (Dako Clone LE-CD19 ref GA656) and anti-GZMB (Dako Clone GrB-7 ref M7235) antibodies according to the manufacturer’s protocols for FFPE biopsies (Akoya Biosciences, Marlborough, MA, USA). Kidney biopsies from patients with (1) stable function without rejection or other significant lesions (STA, *n* = 4), (2) mixed rejection (*n* = 4), and (3) plasma-cell-rich rejection following cessation of immunosuppression (*n* = 4) were used. This rejection is defined by >10% of the plasma cells infiltrating the graft. We also obtained biopsies from one tolerant patient (TOL). This tolerant patient, who underwent transplantation in 2005 and who had been off of his treatment for several years, was biopsied in 2023 following an increase in his proteinuria (1 g/g). His tolerance was confirmed by the results of his biopsy (g0, i1, t0, v0, cpt0, cg0, ci0, ct1, cv0, ah0, mm0, ti0, i-IFTA0, C4d0). This proteinuria, which was associated with a possible infectious episode, returned to its baseline level (0.1 g/g) at 6 months. The data were acquired using an A1R-S confocal microscope (Nikon, Tokyo, Japan). Images were analysed with ImageJ, v2.14.0/1.54f [50].

## 3. Results

### 3.1. Clinical Characteristics of the Patients

The clinicopathological characteristics of the transplant patients who were included in this study are summarised in Table 1. Patients were selected according to gender and age. STA and ABMR patients received standard maintenance immunosuppressive therapy, combining calcineurin inhibitors, antiproliferative agents, and corticosteroids at the time of sampling. STA and TOL patients had normal kidney function, as indicated by their creatininemia and proteinuria.

### 3.2. Natural GZMB^+^ B Cells Display a Differentiated B Cell Profile Enriched in Specific Genes Associated with NK Cells and Regulatory Functions

**Figure 1:**
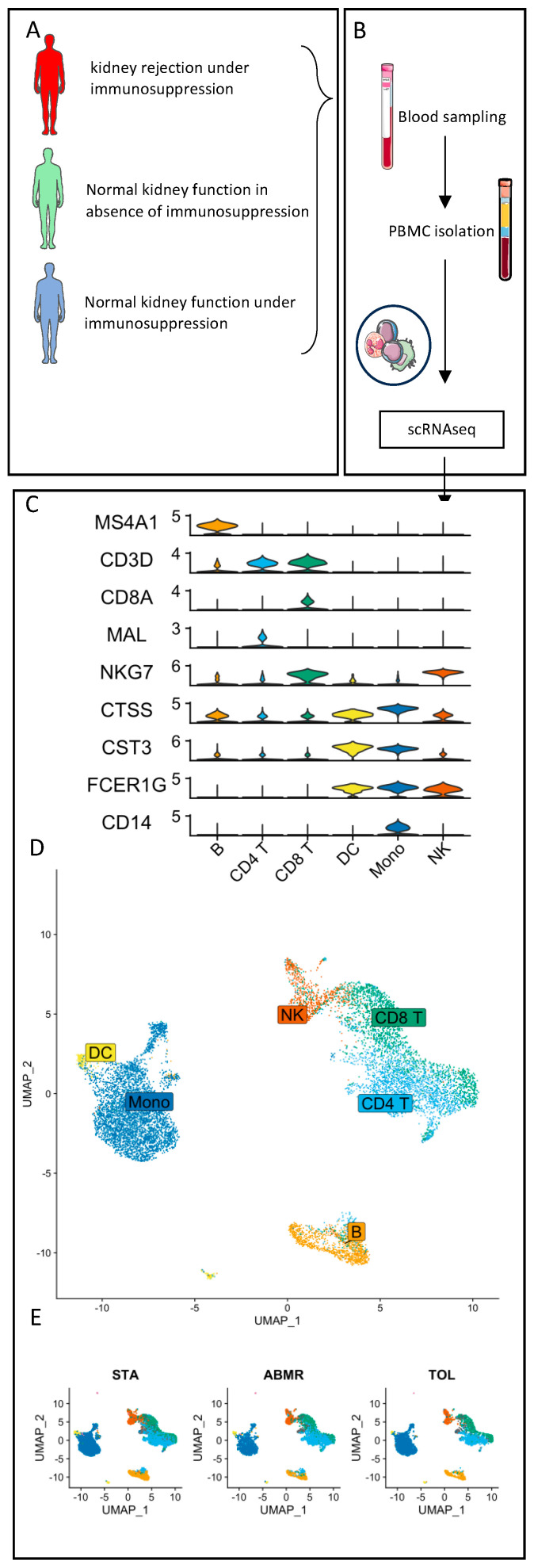
Single-cell RNA sequencing of PBMC from kidney-transplanted patients. Schematic representation of the experiment. (**A**) scRNAseq was performed on PBMCs from kidney-transplanted patients (STA = 3/TOL = 3/ABMR = 3). (**B**) Identical sampling methods were performed for all patients. PBMCs were sequenced using multiplexed CITE-seq protocols. (**C**) Genes identifying immune cell sub-populations are shown in violin plots. (**D**,**E**) UMAP represents the main populations of NK/CD4 T cells/CD8 T cells/DC/monocytes and B cells in blood from transplanted patients. Each dot represents a cell, and each group of coloured dots represents one cell population. UMAPs show all cells of the dataset (**D**) or are split according to the group of patients (**E**).

**Figure 2:**
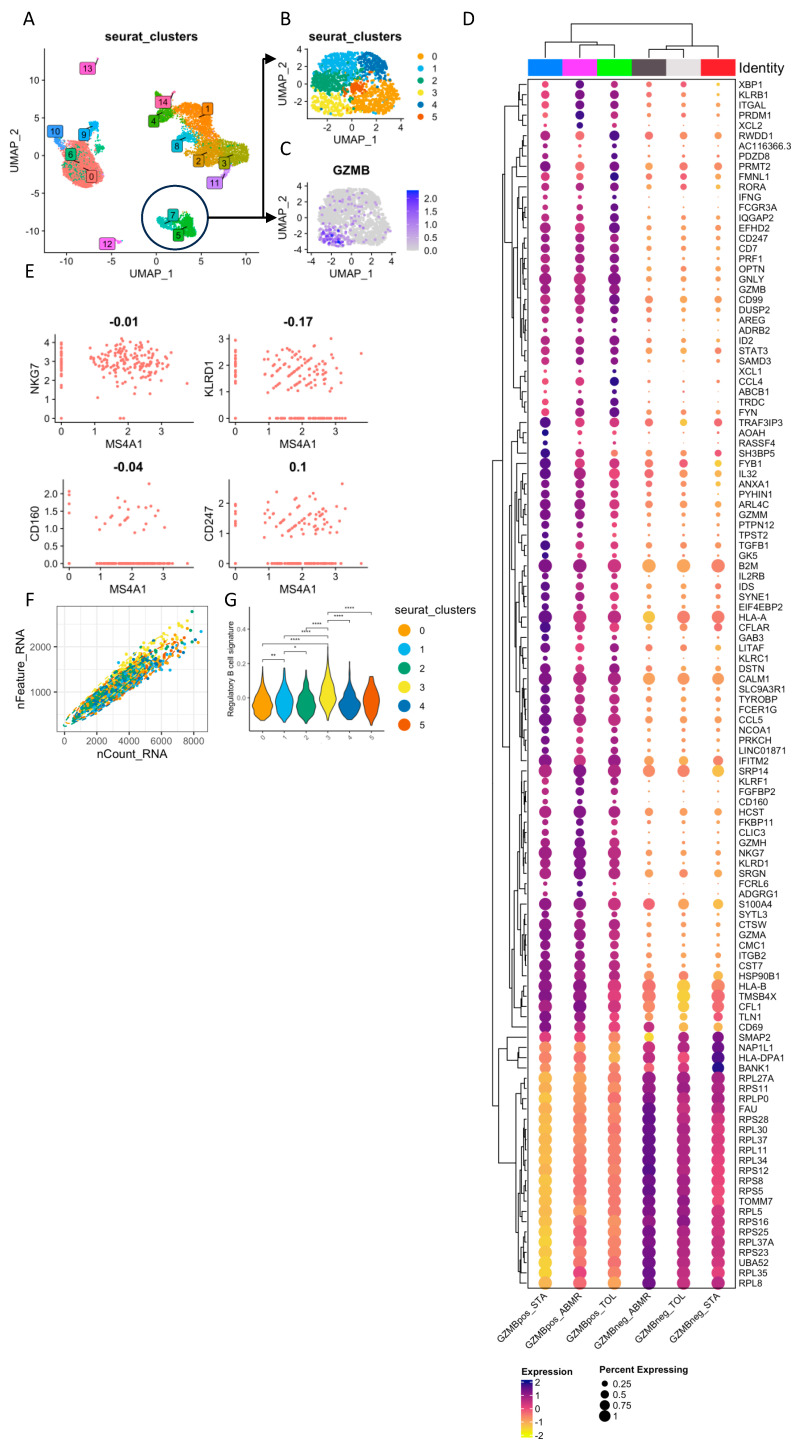
Single-cell sequencing gene expression across B cell clusters. (**A**) UMAP representation of PBMC clusters made using seurat FindClusters() function. Clusters 5 and 7 were associated with MS4A1^+^ B cells. (**B**) UMAP representation of the PBMC Clusters 5 and 7 sub-clusterization leading to the identification of 6 B cell sub-clusters. (**C**) UMAP of GZMB expression in B cells. Cluster 3 was identified as GZMB^+^ B cells. (**D**) Dotplot representation of DEG in GZMB^+^ B cell Cluster 3 and GZMB^−^ B cell Clusters 0, 1, 2, 4, and 5. Only the DEGs within each group of patients are shown. Dots are coloured based on the average expression of the gene in the cluster, and the dot size represents the percentage of cells expressing the gene. (**E**) Scatter plots showing the co-expression of *MS4A1* (CD20) with either *NKG7*, *KLRD1*, *CD160*, or *CD247* in Cluster 3. (**F**) Scatter plot of the quality control metrics “nFeatures_RNA” and “nCounts_RNA” used to determine cell doublets. (**G**) Aggregated average expression levels of each gene of the regulatory B cell signature described by Dubois et al. [37] at the single-cell level, subtracted by the aggregated expression of 100 control features, within B cell clusters. Differences were defined as statistically significant when *p* < 0.01 (*), *p* < 0.001 (**), and *p* < 0.0001 (****).

To characterise GZMB^+^ B cells in the blood of kidney transplant patients, we performed scRNA-seq analysis on total PBMCs, including natural GZMB^+^ B cells, from three TOLs, three STAs, and three ABMRs (Figure 1A,B). A total of 11,432 cells passed quality control for all of the samples, and cell annotation demonstrated the major subpopulations of immune cells, including B cells (MS4A1^+^), CD4 T cells (CD3D^+^, CD8A^−^ and MAL^+^), CD8 T cells (CD3D^+^ and CD8A^+^), NK cells (NKG7^+^, CD3D^−^, and FCER1G^+^), dendritic cells (CTSS^low^, CST3^+^, and FCER1G^+^) and monocytes (CTSS^high^, CD14^+^, and FCER1G^+^) (Figure 1C). In all three patient groups, B cells were well-separated from other immune cell subtypes (Figure 1D,E). B cells were associated with Clusters 5 and 7, using Seurat’s unsupervised clustering. These two B cell clusters were re-clustered into six subclusters for a total of 1405 B cells (Figure 2A,B). Among them and for each group of patients, Cluster 3 (191 cells) expressed the highest level of GZMB and thereby corresponded to GZMB^+^ B cells (Figure 2C). The other groups of B cells were defined as TCL1A^+^ IgM^+^ IgD^+^ naive B cells (Cluster 2), TCL1A^−^ IgA^+^ memory B cells (Cluster 4), or intermediate-stage B cells (Clusters 0, 1, and 5) (Figure S1). Differentially expressed genes (DEGs) were first analysed between Cluster 3 (GZMB^+^ B cells) and the other five clusters (GZMB^−^ B cells) to identify genes specific to these two GZMB^+/−^ B cell populations, regardless of patients’ clinical status. We observed 114 DEGs that were common to all groups of patients between Cluster 3 and all other B cell clusters (FDR < 0.05, |Log2(FC)| > 0.2) (Figure 2D). Cluster 3 expressed high levels of markers, such as *XBP1*, *PRDM1*, and *FKBP11*, which are associated with differentiated memory/plasmablast cells. It also expressed high levels of cytokines and chemokines (*TGFB1*, *IFNG*, *IL32*, *CCL4*, *CCL5*, *XCL1*, and *XCL2*) and markers associated with NK cells (*NKG7*, *KLRD1*, *CD160*, and *CD247*). As *GZMB* is highly expressed in NK cells, we verified that these markers were associated with cells expressing the B-cell-specific marker *MS4A1* (which encodes CD20) and that these B cells met the quality control requirements for the study (Figure 2E,F). This analysis confirmed the absence of cell doublets in Cluster 3 and the absence of contaminating NK cells within the B cells. Finally, we showed a significant enrichment of a “regulatory B cell signature” derived from a bulk RNA-seq meta-analysis, including genes like *GZMB*, *IL10*, and *CCL4* in B cell Cluster 3 [37] (Figure 2G). Overall, these data showed that natural GZMB^+^ B cells, regardless of patient clinical status, were associated with a differentiated gene signature and the expression of genes associated with NK cells and the regulatory B cell signature.

### 3.3. Natural GZMB^+^B Cells Are Highly Differentiated B Cells That Follow a Specific Trajectory Distinct from That of Conventional Memory B Cells, with the KLF13 Gene Overexpressed Throughout This Trajectory

**Figure 3:**
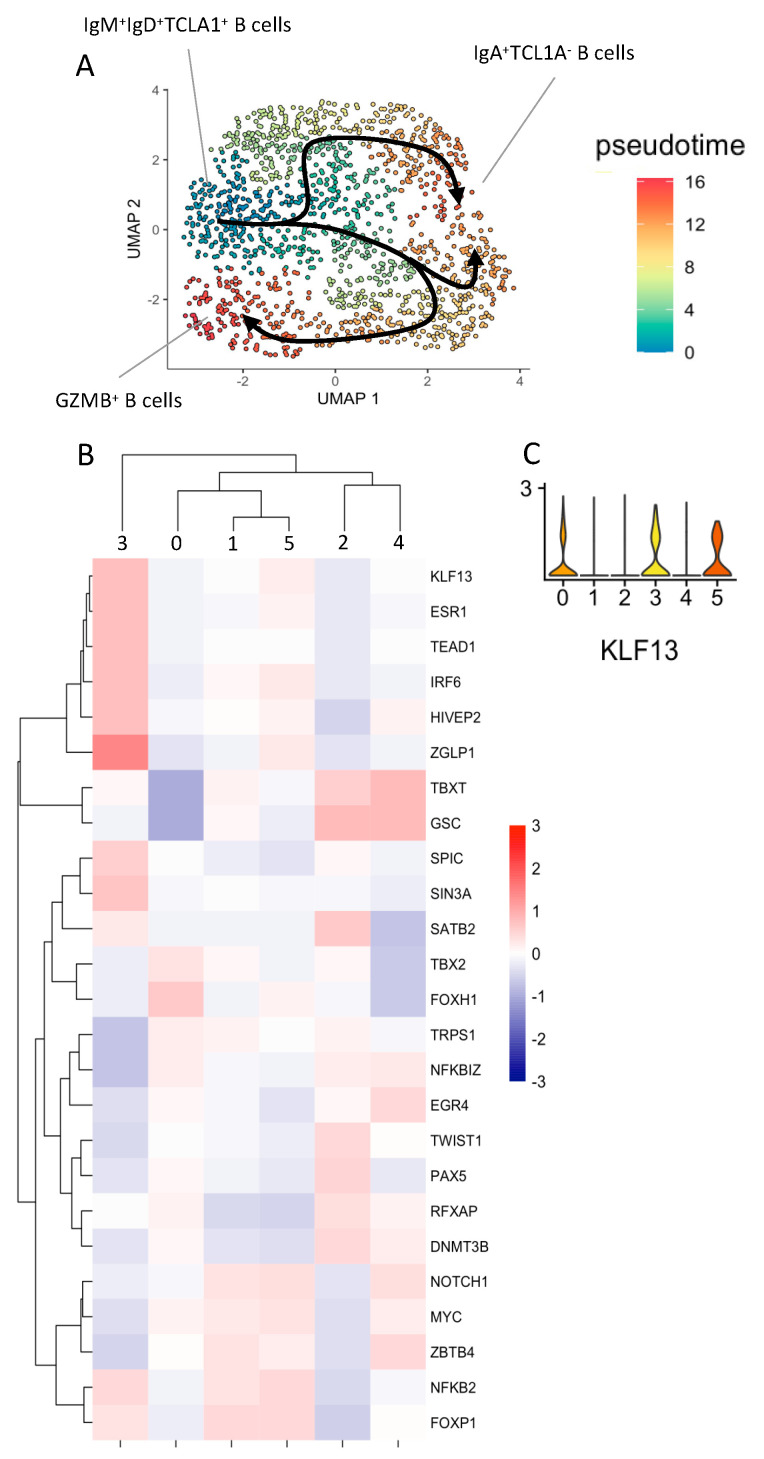
Single-cell RNA sequencing trajectory analysis of B cells: (**A**) UMAP of B cell trajectories using Monocle v3. TCL1A^+^ IgD^+^ IgM^+^ B cells were chosen as the origin cell cluster, and the arrows highlight the trajectories. Cells are coloured according to their differentiated state, ranging from blue (naive) to red (terminally differentiated). (**B**) Heatmap of the significantly enriched target gene sets’ downstream transcription factors grouped according to the B cell clusters. (**C**) Violin plot of *KLF13* expression across B cell clusters.

Given the differentiated profile of GZMB^+^ B cells that was previously shown at the phenotypic level [22] and at the transcriptional level in this study, we compared the trajectories of Cluster 3 and Cluster 4 classical memory B cells (which were identified by low expression of *TCL1A*, *IGHD*, and *IGHM* and high expression of *IGHA1*, *IGHA2*, *IGHG1*, *IGHG2*, and *IGHG3*). Cluster 2, which was associated with naive B cells, was selected as the starting point for both trajectories. Interestingly, we identified two different trajectories starting from Cluster 2 of naive B cells and leading either to Cluster 3 of GZMB^+^ B cells or to Cluster 4 of classical memory B cells (Figure 3A). We then identified transcription factors and associated regulated transcripts that were specific to the B cell clusters along the two trajectories by using the multivariate linear model of DecoupleR. Interestingly, Cluster 3 was specifically associated with increased transcription of genes downstream, KLF13, ESR1, TEAD1, IRF6, HIVEP2, ZGLP1, SPIC, and SIN3A (Figure 3B), with only the *KLF13* gene overexpressed throughout the trajectory of Cluster 3, namely Clusters 0, 3, and 5 (Figure 3C). Overall, these data showed that GZMB^+^ B cells (Cluster 3) were highly differentiated and dissociated early in their differentiation program, with specific transcription factors and a trajectory that were distinct from that of conventional memory B cells.

### 3.4. In General, GZMB^+^ B Cells from Tolerant Patients Exhibit a Specific Transcriptomic Profile Associated with a Decrease in the Expression of HLA Molecules, Apoptosis, and the Inflammatory Response

**Figure 4:**
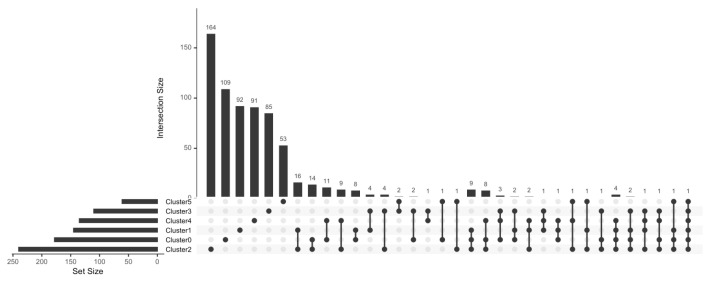
Characterisation of TOL GZMB^+^ B cells’ specific genes. DEGs in TOL patients compared to STA and ABMR within each B cell cluster were summarised in an UpSetPlot representing the number of DEGs in different B cell clusters. Solid black dots represent clusters, and genes differentially expressed in several B clusters are indicated by two or more dots connected by a line. The vertical bar plot indicates the number of DEGs representing each combination, while the horizontal bar plot indicates the number of DEGs between TOL and other groups in each B cell cluster.

GZMB^+^ B cells are more numerous in TOL patients [22]; thus, we analysed the RNA-seq profile of TOL-specific Cluster 3 compared to the profiles of STA and ABMR Cluster 3. A total of 110 DEGs were identified and associated with TOL Cluster 3 (35 genes were upregulated, and 75 were downregulated). To exclude genes associated with all B cells from tolerant patients and not just GZMB^+^ B cells, we excluded DEGs between the TOL and STA/ABMR groups in other B cell clusters. Of these 110 DEGs, 25 genes were shared with other TOL B cell clusters, and 85 genes were found only and specifically in Cluster 3 (Figure 4 and Figure S2). Interestingly, these 85 genes were associated with a decrease in the expression of type II HLA molecules (*HLA-DRA*, *HLA-DRB1*, and *HLA-DPB1*), apoptosis (*WDR26*, *DDX46*, *PIN1*, and *NLRP1*), and the inflammatory response, in general (*ALOX5AP*, *BACH2*, *DPP8*, *CD244*, and *LAMTOR2*).

### 3.5. Ex-Vivo-Induced GZMB^+^ B Cells Share a “Regulatory” Signature with Natural GZMB^+^ B Cells in Tolerant Patients

**Figure 5:**
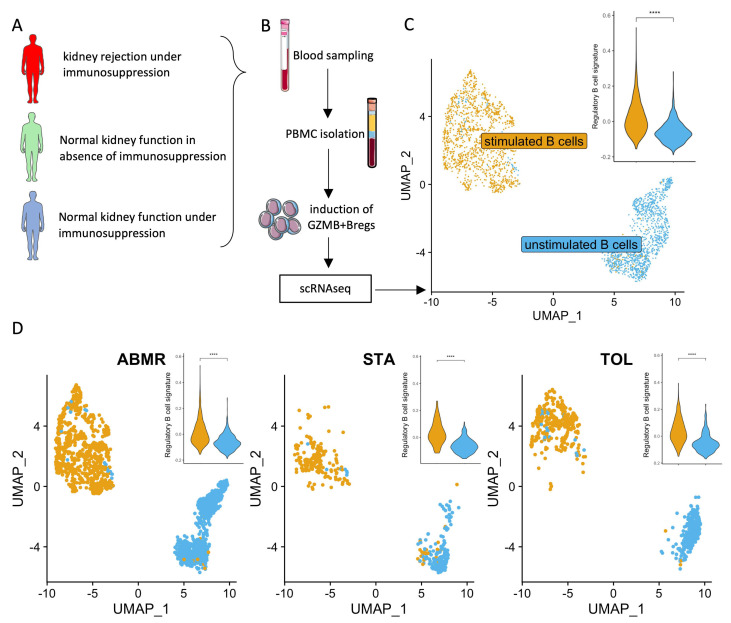
Single-cell RNA sequencing from GZMB^+^ B cells generated in vitro. (**A**) Schematic representation of the groups. RNA sequencing was performed on ex-vivo-induced GZMB^+^ B cells from kidney-transplanted patients (STA, TOL, ABMR) prior to scRNAseq. (**B**) GZMB^+^ B cells or GZMB^−^ B cells were generated from sorted blood B cells for 3 days prior to scRNAseq. (**C**,**D**) UMAP representing the clustering of B cells according to the experimental design in all groups and per group, and violin plot showing the aggregated average expression levels of each gene of the regulatory B cell signature described by Dubois et al. [37] at the single-cell level, subtracted by the aggregated expression of 100 control features within B cell clusters. Each dot represents a cell, and each colour represents either the GZMB^−^ B cells (unstimulated B cells) or the GZMB^+^ B cells (stimulated B cells) across UMAPs. UMAPs show all cells of the dataset (**C**) or are split according to the clinical groups (**D**). Differences were defined as statistically significant when *p* < 0.0001 (****).

**Figure 6:**
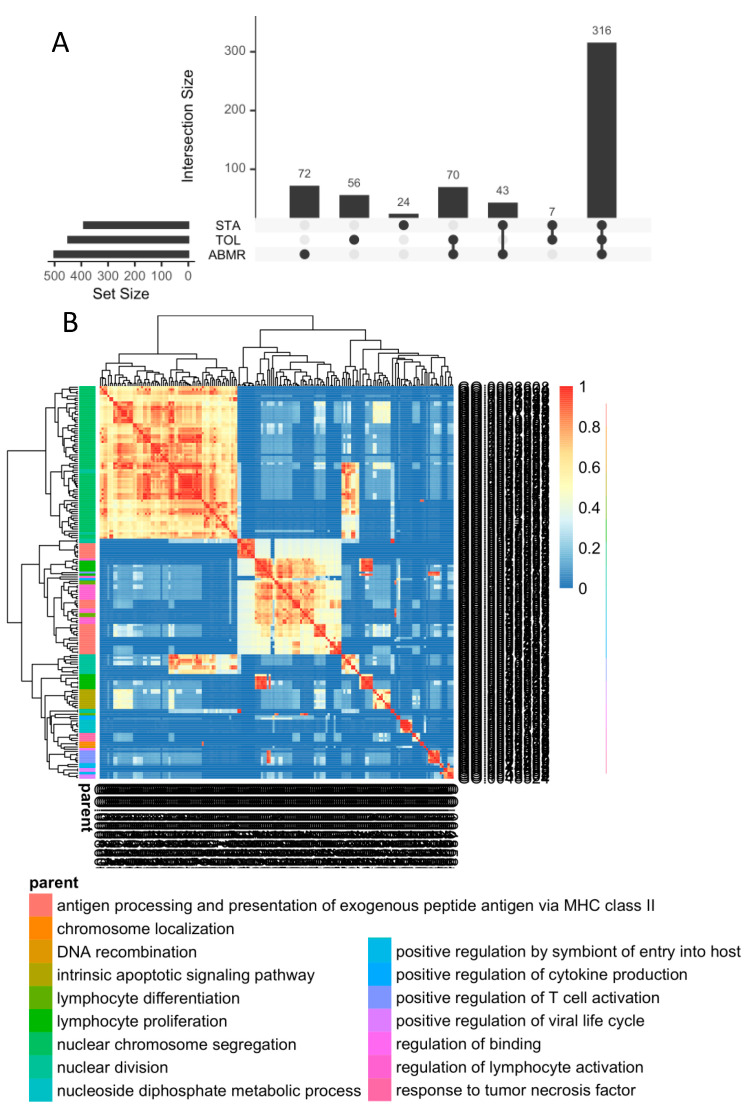
DEG specific to induced GZMB^+^ B cells from TOL. (**A**) DEGs in GZMB^+^ B cells compared to GZMB^−^ B cells within groups were summarised in an UpSetPlot with the number of DEGs between GZMB^+^ and GZMB^−^ B cells. Solid black dots represent groups, and genes differentially expressed in several groups are indicated by two or more dots connected by a line. The vertical bar plot indicates the number of DEGs representing each combination, while the horizontal bar plot indicates the number of DEGs between GZMB^+^ and GZMB^−^ B cells in each group. (**B**) Similarity matrix of the 198 ontologies associated with the 316 differentially expressed genes common to all groups.

We have previously shown that GZMB^+^ B cells can be induced ex vivo from total B cells (>95% purity) [19]. To compare the transcriptomes of induced GZMB^+^ B cells and natural GZMB^+^ B cells from transplanted patients, total B cells from TOLs (*n* = 1), STAs (*n* = 1), and ABMRs (*n* = 2) were cultured for 3 days to induce ex vivo GZMB^+^ B cells or not (GZMB^−^ B cells) (Figure 5A,B). A total of 2603 cells, including 1400 ex-vivo-induced GZMB^+^ B cells and 1203 GZMB^−^ B cells, passed the quality control protocol. First, for natural GZMB^+^ B cells, ex-vivo-induced GZMB^+^ B cells were generally enriched in the “regulatory B cell signature” [37] compared with GZMB^−^ B cells (Figure 5C,D). We then compared ex-vivo-induced GZMB^+^ B cells and GZMB^−^ B cells among the three groups of patients (STA, TOL, and ABMR). A total of 390 DEGs were found between ex-vivo-induced GZMB^+^ B cells and GZMB^−^ B cells in the STA, as well as 449 DEGs in TOLs and 501 DEGs in ABMRs (FDR < 0.05) (Figure 6A). Overall, 316 genes were more closely related to ex-vivo-induced GZMB^+^ B cells than to GZMB^−^ B cells (Figure 6A and Figure S3), with significant enrichment of GO related to the activation status of these cells (genes of activation [GO ID: 0030098; FDR = 1.17 × 10^−7^], proliferation [GO ID: 0046651; FDR = 1.05 × 10^−7^], and response to TNF [GO ID: 0034612; FDR = 0.000141]) and a general decrease in the expression of genes encoding MHC II molecules (GO ID: 0019886; FDR = 2 × 10^−8^) in ex-vivo-induced GZMB^+^ B cells (Figure 6B). Among the TOLs, 56 genes were DE between ex-vivo-induced GZMB^+^ B cells and GZMB^−^ B cells and were not differentially expressed between GZMB^+^ B cells from other transplanted patients (STA and ABMR) (Figure 6A). Among these genes, 40 were upregulated and 16 were downregulated in ex-vivo-induced GZMB^+^ B cells from TOLs. Some of these genes, which were mainly upregulated, were associated with the functions of these cells (GO ID: 0032609; FDR = 0.001171; *TNF* and *LTA*) and migration capacity (GO ID: 0070098; FDR = 0.000103; *CCL3*, *CCL4*, *CCL4L2*, and *CCR7*).

**Figure 7:**
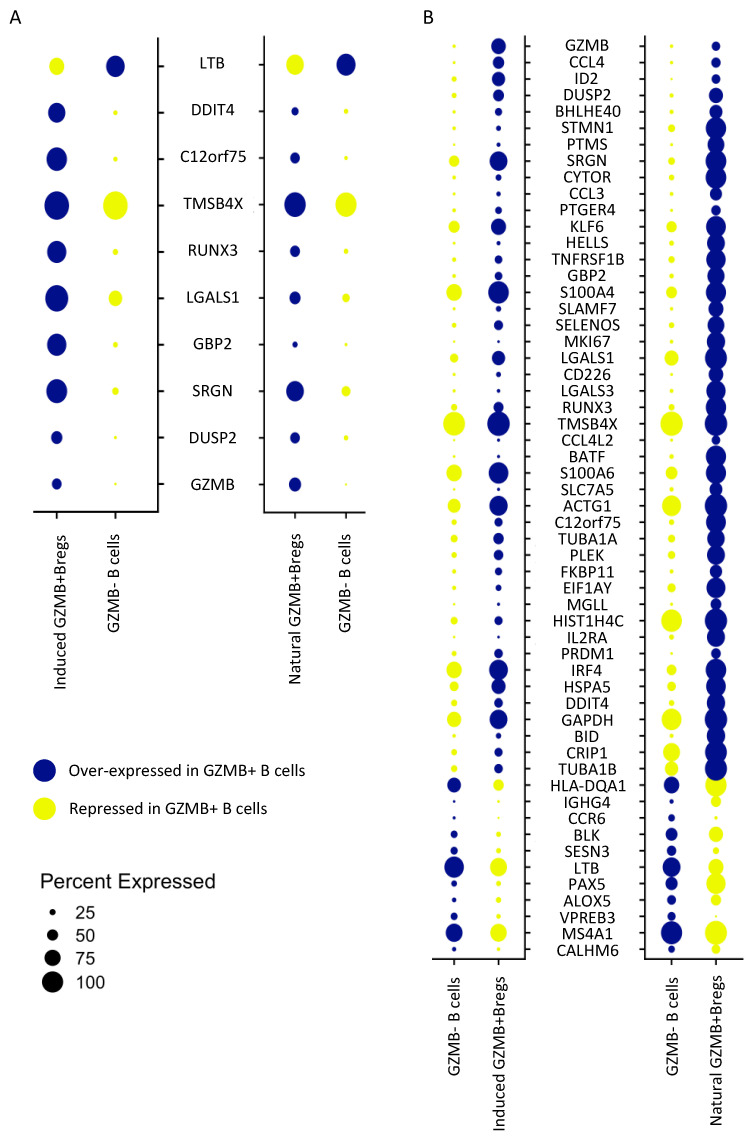
Induced GZMB^+^ B cells’ and natural GZMB^+^ B cells’ gene overlap. (**A**) The signatures of GZMB^+^ B cells generated from the two datasets of induced GZMB^+^ B cells (316 genes) and natural GZMB^+^ B cells (114 genes) have been crossed. The 10 resulting genes are common to natural and induced GZMB^+^ B cells and common to TOL, STA, and ABMR. (**B**) The signatures of GZMB^+^ B cells in TOL generated from the two datasets of induced GZMB^+^ B cells (449 genes) and natural GZMB^+^ B cells (397 genes) have been crossed. The 56 resulting genes are common to natural and induced GZMB^+^ B cells from TOL. The expression levels are shown by dotplots. The width of the dots represents the percentage of expressing cells for each condition. Genes upregulated in GZMB^+^ B cells are associated with blue dots, and genes downregulated in GZMB^+^ B cells are associated with yellow dots.

We subsequently examined the common signatures between ex-vivo-induced GZMB^+^ B cells and natural GZMB^+^ B cells in the different groups of patients. When we examined the signatures that were common to all of the induced or natural GZMB^+^ genes in all patients (316 genes vs. 114 genes, respectively), we observed an overlap of only 10 common genes (Figure 7A). In contrast, when we examined the different groups of patients separately, we observed 56 genes that were common to natural and induced ex vivo GZMB^+^ B cells in the TOLs (Figure 7B), 40 genes in the STA, and 115 genes in the ABMR. Although GZMB^+^ B cells from all of the groups were associated with ontologies related to B cell proliferation and differentiation, only GZMB^+^ B cells from TOLs were enriched in genes related to deletion/regulation (GO ID: 0002683; FDR = 0.003743; *SELENOS*, *IL2RA*, *TNFRSF1B*, *ID2*, *CCL3*, *CCL4*, *SRGN*, *LGALS3*, and *BATF*). These data demonstrate the maintenance of a regulatory profile of GZMB^+^ B cells before and after induction only in TOLs.

Overall, these results show that ex-vivo-induced GZMB^+^ B cells, in addition to expressing a number of genes associated with their inherent status as activated cells, share a “regulatory” signature with natural GZMB^+^ B cells in the TOL.

### 3.6. Natural GZMB^+^ B Cells from Tolerant Patients Interact Strongly with Peripheral Immune Cells

**Figure 8:**
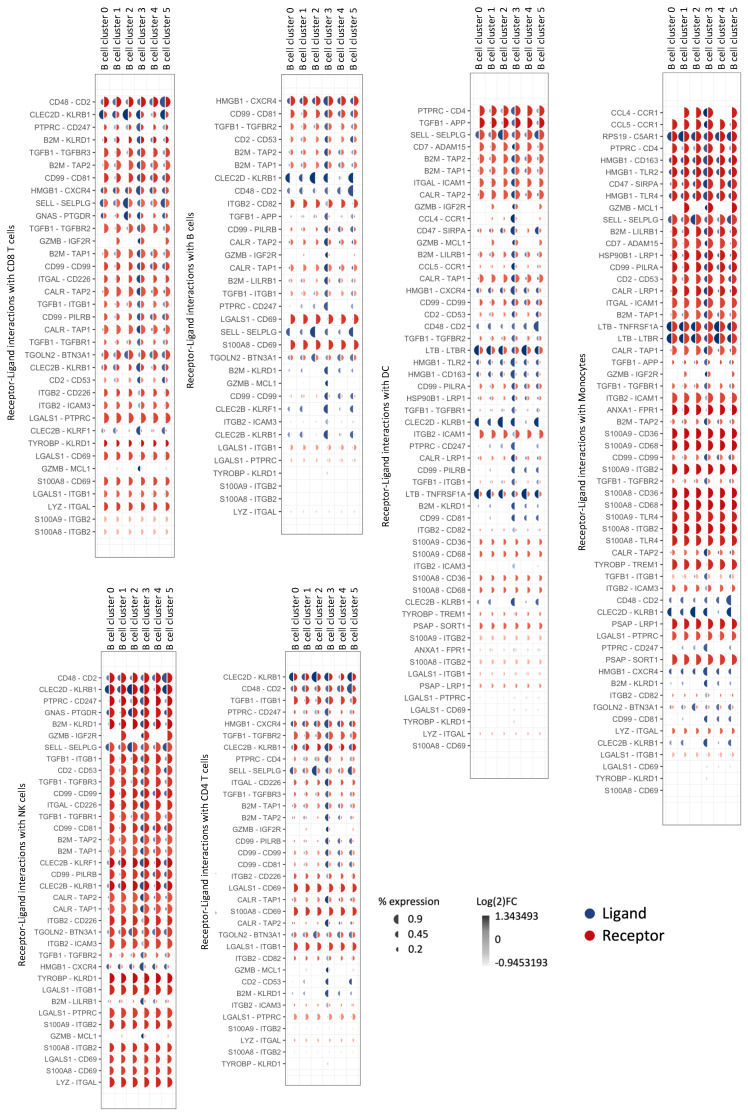
Natural GZMB^+^ B cells ligand–receptor pairs formed with blood immune cells. Nichenet analysis was performed, and communications contributing to signalling from GZMB^−^ and GZMB^+^ B cells to other immune cells are shown in mushroom plots as ligand (blue) and receptor (red) expression across clusters. Each plot is associated with one population of target cells. The size of the semi-circles represents the percentage of cells expressing the gene, and the colour intensity represents the relative expression across B cell clusters (ligand) and immune populations (receptor).

**Figure 9:**
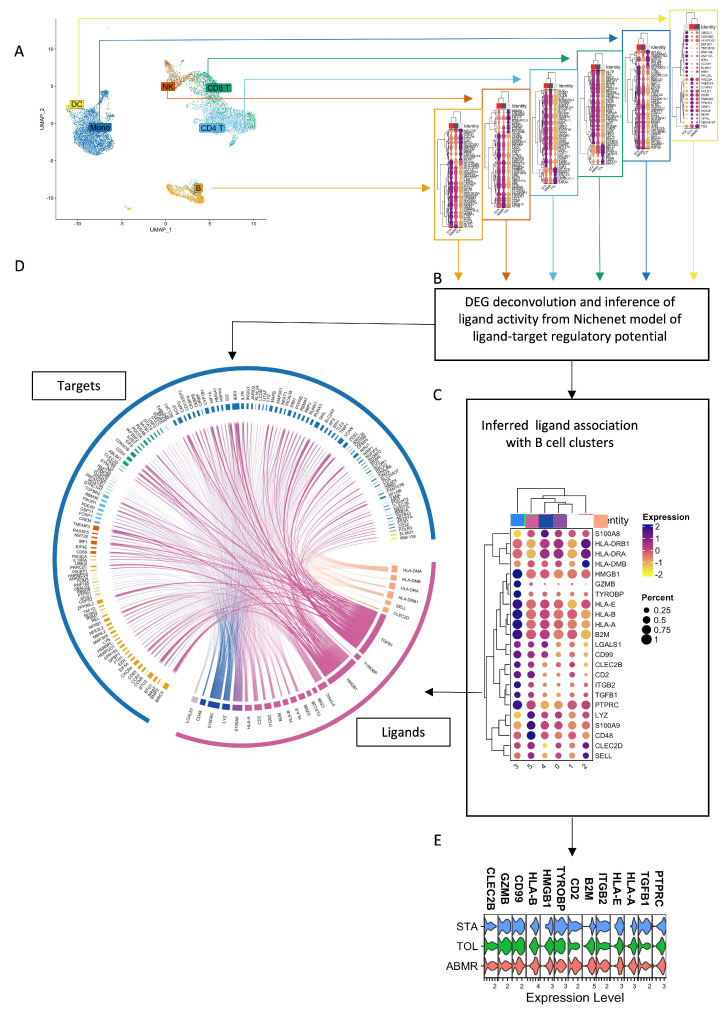
GZMB^+/−^ B cell communication with blood immune cells in TOL. (**A**) DEGs between TOL and STA or ABMR among dendritic cells (yellow), monocytes (dark blue), CD8 T (dark green), CD4 T (light blue), NK (vermilion), and B cells (orange) were used to infer ligand activity based on the Nichenet model. Only the 50 most common DEGs are represented in the dotplots. (**B**) Nichenet uses correlation matrix of ligand–target regulatory potential generated from public databases to infer ligand and receptor activity. (**C**) Ligands predicted to be associated with the transcriptional profile in TOL were then visualised within B cell clusters, as represented with dotplots. (**D**) Circos plot visualisation of predicted ligands on GZMB^+^ and GZMB^−^ B cell clusters and their targets on the different immune populations. The width of the arrows represents the strength of the interactions according to the Nichenet model. Target genes are coloured according to the immune population in which they are differentially expressed (same colours as Figure 7A). Arrows are coloured according to the B cell cluster overexpressing the associated ligand (same colours as Figure 7C). Only the 2000 strongest associations are represented (ligand to target pairs, represented by the arrows). (**E**) Expressions of ligands assigned to GZMB^+^ B cell Cluster 3 are represented by violin plots.

We subsequently examined the predicted interactions of natural GZMB^+^ B cells or GZMB^−^ B cells with B cells, T cells (CD4^+^ and CD8^+^ T cells), NK cells, dendritic cells, and monocytes from the PBMCs of transplanted patients (TOL, STA, and ABMR) by using Nichenet. A total of 40 unique ligands were expressed on B cells (GZMB^+^ and GZMB^−^ indifferently), and their receptors were expressed on target cells. Of these, 17 ligands were associated with different clusters of GZMB^−^ B cells (Clusters 0, 1, 2, 4, or 5), and 22 ligands were specifically associated with Cluster 3, with only 1 ligand shared by both GZMB^+^ and GZMB^−^ B cells (Figure 8, Table 2). Among them, several GZMB^+^ B cell interactions were associated with interactions involved in known regulatory mechanisms (CD47-SIRPA, CCL4-CCR1, CCL5-CCR1, TGFB1-TGFBR1 + 2 + 3, and GZMB-IGF2R/MCL1). Overall, 24 of these ligands on GZMB^+^ or GZMB^–^ were specifically associated with a significant enrichment of target genes in TOLs compared with the other groups (FDR < 0.05) (Figure 9A–D). Among these ligands, 13 ligands (*GZMB*, *TGFB*, *HLA-A*, *HLA-B*, *HLA-E*, *PTPRC*, *TYROBP*, *HMGB1*, *B2M*, *CD2*, *ITGB2*, *CD99*, and *CLEC2B*) were enriched in GZMB^+^ B cells only. Interestingly, whereas the expression of the 13 ligands was equivalent among groups within the GZMB^+^ B cell cluster (Figure 9E), these 13 ligands were predicted to be associated with a significant enrichment of target genes in TOLs, with 251 targets present in TOLs only; moreover, 48 target genes were present on B cells (23% of B cell DEGs between TOLs and STAs/ABMRs), 16 target genes on NK cells (20%), 4 target genes on DCs (16%), 100 target genes on monocytes (29%), 43 target genes on CD4 T cells (19%), and 43 target genes on CD8 T cells (15%). Overall, these results demonstrated an enrichment of interactions between natural GZMB^+^ B cells and PBMCs, especially in blood from TOLs, compared with that in blood from other patients.

### 3.7. Under Inflammatory Conditions, GZMB^+^ B Cells Can Infiltrate Kidney Allografts

**Figure 10:**
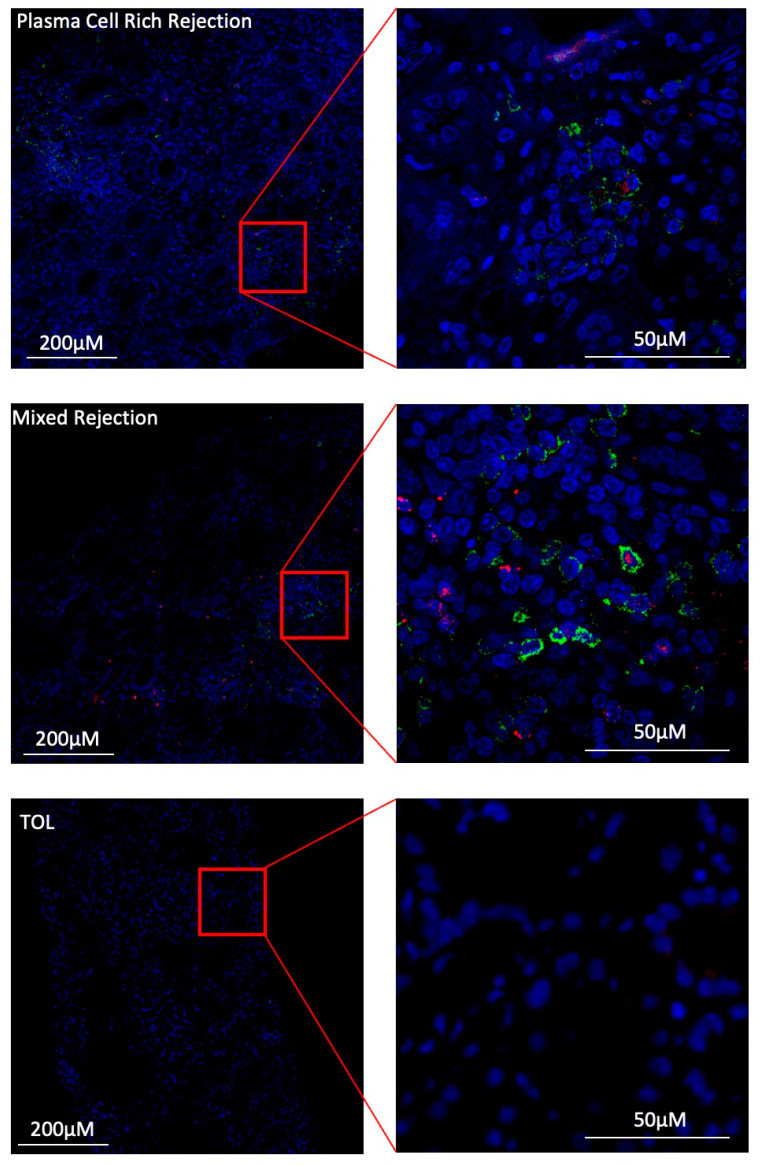

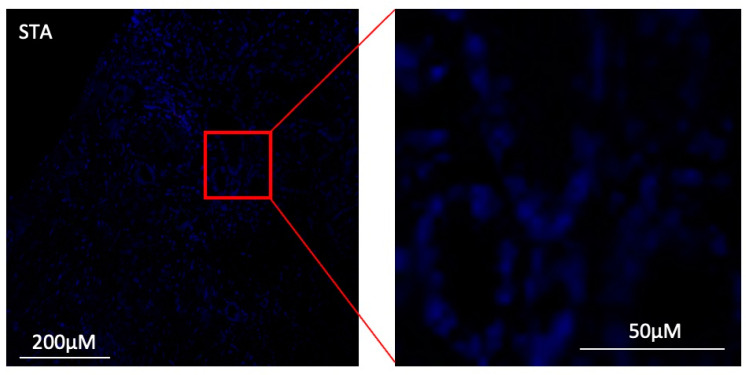
GZMB^+^ B cells infiltrate the graft under inflammatory conditions. IHC staining using the OPAL multiplex system in one representative biopsy of patient with plasma-cell-rich rejection (first row), mixed rejection (second row), tolerance (third row), and stability (last row) at 50 and 200 µM. Enlargement of one representative CD19^+^ GZMB^+^ cell was performed for all patients. For all images, the 3 fluorescence channels are merged to form one picture coloured by canal (DAPI—blue; CD19—green; GZMB—red).

To date, only GZMB^+^ B cells in the blood compartment of kidney transplant patients have been investigated. We analysed the presence of GZMB^+^ B cells in renal biopsies from STA (*n* = 4), mixed rejection (*n* = 4), plasma-cell-rich rejection (*n* = 4), and TOL (*n* = 1) patients. As expected, no immune cell infiltrates were present in the biopsies of the STA or TOL patients with good and stable graft function. Biopsies from patients with plasma-cell-rich rejection and mixed rejection were characterised by a large infiltrate of CD19^+^ B cells in the presence of slight and local CD19^+^ GZMB^+^ B cells (Figure 10). Interestingly, these results suggest that GZMB^+^ B cells may infiltrate the graft under inflammatory conditions, such as in mixed rejection and plasma-cell-rich rejection conditions.

## 4. Discussion

We have shown that patients who tolerate their kidney grafts harbour B cells with regulatory properties in which the mechanisms are dependent on the granzyme B molecule and on contact between the GZMB^+^ cells and their target [20,36,39]. We have also shown that such GZMB^+^ B cells are present in the blood of healthy donors and that, despite their rarity in vivo, they can be induced ex vivo with IL-21, IL-2, CpG DNA, CD40L, and BCR agonists [19,35]. Thus, these GZMB^+^ B cells are promising candidates for cell therapy in various diseases in which such regulatory processes are lacking, as well as in solid organ transplantations in which GZMB^+^ B cells are not only more numerous in patients who tolerate their kidney grafts [31] but also have been shown to transfer allograft tolerance in a rat model of heart transplantation [18].

In recent years, numerous studies have reported Bregs with different phenotypes and mechanisms in animal models [5,51,52] and in humans [35,53,54,55,56]. Based on these studies, we know that Bregs have multiple phenotypes and functions that may vary according to the method of stimulation, pathology, model, or even microenvironment within the same subject [9,51]; additionally, to date, no common phenotype has been reported for these cells [37]. Different transcription factors have been associated with Bregs depending on the tissue in which they are found [51], and it has been shown (notably, for B10 cells) that these cells can emerge at different stages of maturation from B cells [9], thus leading to questions concerning their development and unique origin. Finally, it remains unclear as to whether these cells are the same in blood from healthy volunteers or in specific situations, such as transplantation, and/or whether they may acquire specific functions. This question remains unresolved in the context of tolerance, wherein they appear to be more numerous and able to prevent T-cell proliferation in a similar manner as Bregs from healthy volunteers or other patients transplanted in different clinical situations [22]. However, their specific transcriptomic profile and their capacity to interact with other immune cells remain unknown.

To answer this question, we performed a single-cell RNA-seq analysis of PBMCs from kidney transplant patients (STA, ABMR, and TOL) with a focus on GZMB^+/−^ cell clusters. The objective of this study was to identify whether there was a specific profile of GZMB^+^ B cells compared with that of GZMB^−^ B cells in transplanted patients, particularly in tolerant patients, when GZMB^+^ B cells were directly sorted from blood without any in vitro restimulation. We also compared the profiles of these GZMB^+^ B cells from tolerant patients with those of GZMB^+^ B cells from other transplanted patients and analysed their trajectories and interactions with other immune cells, particularly in tolerant patients. Finally, we compared the profiles of GZMB^+^ B cells before and after ex vivo induction and analysed these GZMB^+^ B cells in the grafts of transplant patients in different clinical situations.

We showed that B cells expressing GZMB can be easily distinguished from other B cells on the basis of their highly differentiated profile, their expression of regulatory genes, such as *TGFB1* and *IL2RA* [16,20,57], and their expression of several NK markers, such as *NKG7*, *KLRD1*, *CD160*, and *CD247*. Interestingly, whereas this signature of NK-B cells has been demonstrated at the onset of inflammation, such as SIV/HIV infection [58,59], wherein GZMB^+^ B cells were also described [36,60], this tolerance phenotype of NK-B cells is supported by a recent study showing increased CD56 expression on B cells in the blood of a cohort of tolerant patients after renal transplantation [61].

Due to the fact that this population is rare, the absolute number of GZMB^+^ B cells in the dataset was low, thus making it more difficult to identify differential genes between these GZMB^+^ B cell subpopulations. Nevertheless, among the genes that were specifically expressed in GZMB^+^ B cells, 85 are specific to TOLs and shared neither by other B cell subtypes nor by the GZMB^+^ B cells of the STA or ABMR, with a decrease in the number of genes encoding HLA molecules (*HLA-DRA*, *HLA-DRB1*, and *HLA-DPB1*), apoptosis (*WDR26*, *DDX46*, *PIN1*, and *NLRP1*), and the inflammatory response in general (*ALOX5AP*, *BACH2*, *DPP8*, *CD244*, and *LAMTOR2*). Interestingly, this signature, which we previously identified in GZMB^+^ B cells from healthy donors [35], is also a well-known mechanism of immune evasion aimed at reducing the activation capacity of T cells [62].

We also showed that GZMB^+^ B cells are highly differentiated B cells. These data corroborate what we had already shown at the protein level with cells expressing markers of activation and differentiation (XBP1, PRDM1, and FKBP11) [63,64]. Interestingly, this “differentiated” profile that is shared by many Bregs (regardless of their function) in the absence of a consensus marker [9,65,66,67] is also associated with GZMB^+^ B cells, which have a unique trajectory that is different from that of classic switch memory cells, with specific expression of certain transcriptional markers that are absent from the trajectory of classic switch memory cells, such as KLF13. These data suggest that these GZMB^+^ B cells do not emerge from B cells at different stages of differentiation, as has been shown for B10 cells [9]; rather, they originate from a specific lineage, which is a finding that is newly discovered in the field of Bregs. Interestingly, KLF13 is a transcription factor in which the induction is associated with an improvement in the symptoms of sepsis-induced myocardial injury in a mouse model [68]. KLF13 is also involved in several stages of lymphoid cell development [69]. Currently, there is no evidence specifically associating this KLF13 transcription factor with Bregs. However, one of its targets is CCL5 [70], which is a chemokine that has been found to be upregulated in GZMB^+^ B cells and corresponds well to the migratory transcriptional profile that is also found in these cells.

Another objective of this study was to compare the transcriptomic profile of natural regulatory GZMB^+^ B cells with that of ex-vivo-induced GZMB^+^ B cells when considering that after culture, ex-vivo-induced GZMB^+^ B cells retain their suppressive properties [19]. We showed that ex vivo induction of GZMB^+^ B cells is associated with an activation profile, with a general enrichment of the “TNF response” pathway, as has been previously described for healthy volunteers [35]. Some of these molecules are already expressed by natural GZMB^+^ B cells. This is not surprising because, like all Bregs, they require a certain level of activation to express their regulatory properties [21]. The overlap between natural and ex-vivo-induced GZMB^+^ B cells remained low in all patients. Such discordant gene profiles have already been previously described between Bregs before and after ex vivo induction by K. Wood’s group [67]. Thus, after 7 days of culture, although ex-vivo-induced B10 cells retain their suppressive properties, these cells act through different mechanisms, with a shift from IL-10-dependent to TIM-1-dependent regulation [67]. Although the “transcriptional profile” of GZMB^+^ B cells may differ before and after induction, the suppressive effects of GZMB^+^ B cells are partially dependent on GZMB [19,22]. Nevertheless, we have previously shown that GZMB^+^ B cells from healthy volunteer B cells induced ex vivo act via GZMB, with LTα regulating its expression [35]. LTα, which is a member of the TNF superfamily, is secreted by GZMB^+^ B cells and, through an autocrine mechanism, enhances the expression of GZMB, which is responsible for the regulatory functions of these cells. Among transplant patients, only ex-vivo-induced GZMB^+^ B cells from TOLs overexpressed LTα, thus suggesting that different mechanisms of action may exist according to the origin of GZMB^+^ B cells or their mode of production. These data therefore suggest the closer proximity between GZMB+ cells from TOLs and those from healthy volunteers compared with those from other groups of transplant patients.

These different signatures of GZMB^+^ B cells, depending on the patient’s clinical situation and the level of activation of these cells (natural or induced ex vivo), also strongly suggest a role for the microenvironment in the emergence of such regulatory populations in vivo. These findings are similar to results from the last century showing that, in transplantation, regulatory mechanisms only occur when the graft is present; moreover, if the graft is removed, these “natural” regulatory mechanisms disappear [71]. Therefore, if such a theory is true, we should expect certain common mechanisms of action, such as GZMB, distinct mechanisms between ex-vivo-induced GZMB^+^ B cells and natural GZMB^+^ B cells from healthy volunteers, and distinct mechanisms between GZMB^+^ B cells from patients in different clinical situations after transplantation.

By analysing receptor–ligand interactions, we first showed that GZMB^+^ B cells are more likely to interact with blood immune cells other than GZMB^−^ B cells through several ligand–receptor interactions that have been shown to be fundamental in the regulation of the immune response, such as CD47-SIRPA [72], CCL4-CCR1 [73,74], CCL5-CCR1 [73], TGFB1-TGFBR1 + 2 + 3 [16,75], and GZMB-IGF2R/MCL1 [32,76]. Interestingly, these receptor–ligand interactions are stronger in tolerant patients, with a greater number of targets downstream of these interactions, which is likely due to the greater frequency of GZMB^+^ B cells in the blood of these patients [22]. GZMB^+^ B cells interact mainly with monocytes and B cells, which are two populations that are known to strongly infiltrate inflammatory grafts [77,78,79,80,81,82,83,84,85,86,87]. These data are consistent with the finding that natural GZMB^+^ B cells also express molecules involved in migration, such as the chemokine receptor CX3CR1, in which its ligand (CX3CL1) is expressed by renal endothelial cells under inflammatory conditions [88]. Similarly, after activation, ex-vivo-induced GZMB^+^ B cells also overexpress the chemokines CCL4 and CCL5, which have been shown to attract [89] and inhibit IL-1β secretion by monocytes [73].

This migratory profile of GZMB^+^ B cells raises another important question as to whether these circulating cells can migrate and infiltrate the graft where immune events occur. Few studies have examined the graft environment in tolerant patients for various reasons, including the rarity of these patients and ethical reasons, as patients are not biopsied in the absence of suspected rejection. Thus, the few data generated from biopsies of tolerant patients are not very clear and can vary depending on the strength of the focal interstitial infiltrate [90,91,92,93,94]. We observed no infiltration in the biopsies of the TOLs and STAs, which is consistent with their stable graft function. In contrast, we detected B cells and GZMB^+^ B cells in kidney biopsies from transplanted patients with signs of rejection (mixed and plasma-cell-rich rejections), thus suggesting that GZMB^+^ B cells can infiltrate the graft under inflammatory conditions. Similarly, it was recently shown that GZMB^+^ B cells were able to infiltrate liver grafts in patients with intrahepatic cholangiocarcinoma [95]. Although the presence of infiltrating regulatory T cells has been demonstrated, even with the identification of some specific populations of memory regulatory T cells [4,96], this has not yet been demonstrated for Bregs in humans.

## 5. Conclusions

In conclusion, we identified a specific profile for GZMB^+^ cells with a specific differentiation trajectory. In particular, in TOLs, such GZMB^+^ cells displayed increased ligand–receptor interactions and numerous potential downstream targets, thus suggesting the acquisition of a specific phenotype that may be due to the progressive establishment of a protolerogenic environment in these patients. We also demonstrated a conserved regulatory signature between natural GZMB^+^ TOLs and ex-vivo-induced GZMB^+^ TOLs that was highly similar to that of GZMB^+^ cells from other groups. Finally, we reported that these GZMB^+^ B cells can infiltrate the graft under inflammatory conditions, thus acting in locations where the events occur.

## Figures and Tables

**Table 1:** Clinical data associated with the samples used for scRNAseq.

	STA (*n* = 4)	ABMR (*n* = 5)	TOL (*n* = 4)
Sex (M/F)	3/1	2/3	3/1
Age at the time of analysis (years)	46.25 ± 17	55.4 ± 4	45.75 ± 19.1
Donor (LD/NLD)	0/4	1/4	2/2
Time post-transplantation (days)	1056 ± 1381	2178 ± 2783	6891 ± 3709
Creatinemia (μMol)	85.25 ± 23.01	159.6 ± 42.20	126.25 ± 52.81
Proteinuria (g/g)	0 ± 0	0.405 ± 0.276	0.05 ± 0.01
Mismatched HLA *	4/5/5/5	2/4/2/1/3	4/3/0/0
Immunosuppression at sampling **	4	5	0
CNIs	4	5	0
Steroids	2	5	0
Antiproliferative	4	4	0

F: female; M: male; LD: living donor; NLD: non-living donor; * as the quantity of mismatch for each patient; ** as the quantity of patients associated with each immunosuppression category.

**Table 2:** Receptor–ligand pairs associated with each B cell cluster.

Sender B Cell Cluster	Ligand-Receptor Pairs	Target Cells
B cell Cluster 0 (GZMB^−^)	/	/
B cell Cluster 1 (GZMB^−^)	RPS19-C5AR1	Mono
B cell Cluster 2 (GZMB^−^)	HLA-DMA-CD4/HLA-DMA-CD74/HLA-DPB1-CD4/HLA-DRB1-CD37/HLA-DRB1-CD53/HLA-DRB1-CD81/HLA-DRB1-CD82 HLA-DQA1-CD4/SELL-SELPLG/CLEC2D-KLRB1/HLA-DQB1-CD4/HLA-DRA-CD4/HLA-DRA-CD37/HLA-DRA-CD53/HLA-DRA-CD63/HLA-DRA-CD81/HLA-DRA-CD82/HLA-DMB-CD4/HLA-DMB-CD74	CD4 T/CD8 T/NK/Mono/B/DC
B cell Cluster 3 (GZMB^+^)	CLEC2B-KLRF1/CALR-TAP1/CALR-TAP2/CALR-LRP1/TYROBP-TREM1/TYROBP-KLRD1/HLA-F-CD8A/HLA-F-LILRB1/HLA-F-LILRB2/CD99-CD99/HSP90B1-LRP1/ITGB2-ICAM1/ITGB2-ICAM3/ITGB2-CD82/ITGB2-CD226/B2M-TAP1/B2M-TAP2/B2M-LRP1/B2M-LILRB1/CD7-ADAM15/GZMB-IGF2R/GZMB-MCL1/HLA-B-CD8A/HLA-B-LILRB1/HLA-B-LILRB2/HLA-B-KLRD1/HMGB1-TLR2/HMGB1-TLR4/HMGB1-CXCR4/HMGB1-CD163/HLA-E-CD8A/HLA-E-KLRD1/CD2-CD53/ANXA1-FPR1/TGFB1-TGFBR1/TGFB1-TGFBR2/TGFB1-TGFBR3/TGFB1-APP/TGFB1-ITGB1/HLA-A-CD8A/HLA-A-LILRB1/HLA-A-LILRB2/HLA-A-KLRD1/HLA-A-APLP2/HLA-C-LILRB1/HLA-C-LILRB2/PTPRC-CD4/PTPRC-CD247/CD47-SIRPA/CCL4-CCR1/CCL5-CCR1	CD4 T/CD8 T/NK/Mono/B/DC
B cell Cluster 4 (GZMB^−^)	S100A8-CD36/S100A8-CD68/S100A8-CD69/S100A8-ITGB2	CD4 T/CD8 T/NK/Mono/B/DC
B cell Cluster 5 (GZMB^−^)	LYZ-ITGAL/S100A9-CD36/S100A9-CD68/S100A9-ITGB2/S100A9-TLR4/CD48-CD2	CD4 T/CD8 T/NK/Mono/B/DC
General expression	LGALS1-CD69/LGALS1-ITGB1/LGALS1-PTPRC	CD4 T/CD8 T/NK/Mono/B/DC

## Data Availability

All of the data that were generated for this study are available upon reasonable request.

## Supplementary Materials

The following supporting information can be downloaded at: https://www.mdpi.com/article/10.3390/cells13151287/s1, Figure S1: B cell phenotypic markers displayed as violin plots across B cell clusters; Figure S2: TOL-specific genes in natural GZMB^+^ B cells; Figure S3: Dotplot showing differentially expressed genes between GZMB^+^ and GZMB^−^ B cells shared by all groups of transplanted patients.

## Abbreviations

GZMB^+^ B cells:	Granzyme B positive B cells with regulatory properties.
Induced GZMB^+^ B cells:	GZMB^+^ B cells generated according to the in vitro protocol described in Chesneau et al. [19].
Natural GZMB^+^ B cells:	B cells from donor’s blood or biopsies expressing GZMB without any restimulation in vitro.
GZMB^−^ B cells:	B cells used to determine the characteristics of GZMB^+^ B cells. For the dataset generated after in vitro induction, GZMB^−^ B cells correspond to the conditions that did not receive any stimulation (i.e., total resting B cells after 3 days), as GZMB expression is transient.
TOL:	Patients with no immunosuppression for at least one year and with stable creatinine < 150 mmol/L and proteinuria < 1 g/24.
STA:	Patients with stable graft function (creatinine < 150 mmol/L and proteinuria < 0.2 g/g creatinine) for at least 3 years under standard immunosuppression (calcineurin inhibitors [CNIs], antimetabolite ± corticosteroids).
ABMR:	Patients with histology-proven antibody-mediated rejection.
DEG:	Differentially expressed gene.

## Appendix A

DIVAT (Données Informatisées et VAlidées en Transplantation) Consortium, DIVAT Cohort Collaborators (Medical Doctors, Surgeons, HLA Biologists).

***Lyon E. Hériot***: Lionel Badet, Maria Brunet, Fanny Buron, Rémi Cahen, Ricardo Codas, Sameh Daoud, Valérie Dubois, Coralie Fournie, François Gaillard, Arnaud Grégoire, Alice Koenig, Charlène Lévi, Emmanuel Morelon, Claire Pouteil-Noble, Maud Rabeyrin, Thomas Rimmelé, and Olivier Thaunat.

***Nantes***: Gilles Blancho, Julien Branchereau, Diego Cantarovich, Agnès Chapelet, Jacques Dantal, Clément Deltombe, Lucile Figueres, Raphael Gaisne, Claire Garandeau, Magali Giral, Caroline Gourraud-Vercel, Maryvonne Hourmant, Georges Karam, Clarisse Kerleau, Delphine Kervella, Christophe Masset, Aurélie Meurette, Simon Ville, Christine Kandell, Anne Moreau, Karine Renaudin, Florent Delbos, Alexandre Walencik, and Anne Devis.

***Paris-Necker***: Lucile Amrouche, Dany Anglicheau, Olivier Aubert, Lynda Bererhi, Christophe Legendre, Alexandre Loupy, Frank Martinez, Arnaud Méjean, Rébecca Sberro-Soussan, Anne Scemla, Marc-Olivier Timsit, and Julien Zuber.
