# Supplementary material for: Regulatory B Cells Expressing Granzyme B from Tolerant Renal Transplant Patients: Highly Differentiated B Cells with a Unique Pathway with a Specific Regulatory Profile and Strong Interactions with Immune System Cells
Source: Cells. 2024 Jul 31;13(15):1287. doi: 10.3390/cells13151287 (PMC11311295; doi:10.3390/cells13151287)
Supplement: Supplementary file 1 [file cells-13-01287-s001.zip › cells-3050625-supplementary.pdf]

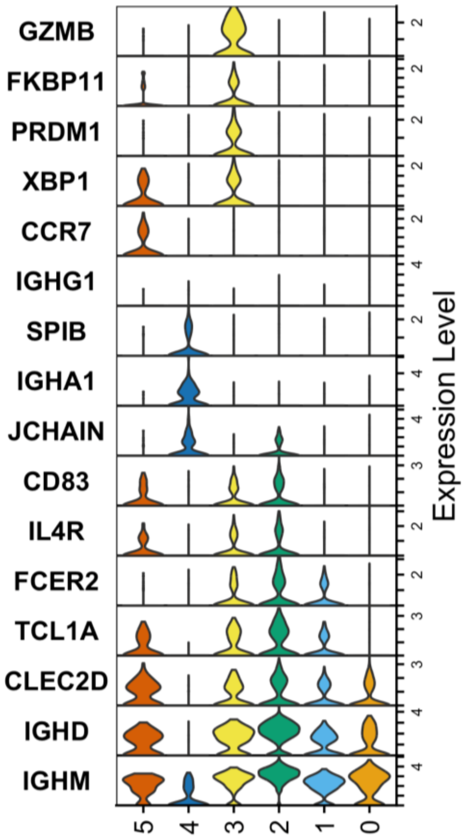

Figure S1: B cell phenotypic markers displayed as violin plots across B cell clusters.

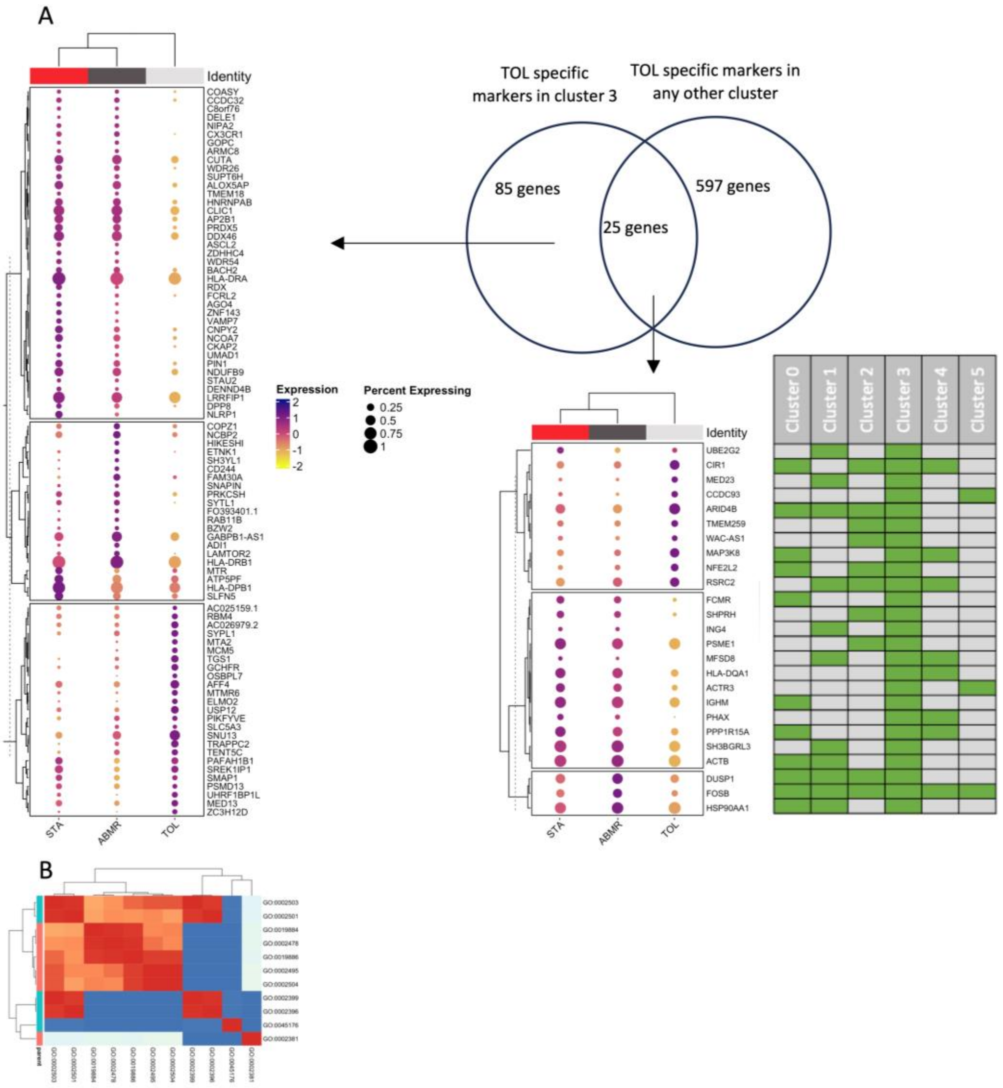

Figure S2 TOL-specific genes in natural GZMB+ B cells: (A) Venn diagram of the DEG between TOL and other groups from cluster 3 (110 genes) and all other clusters (622 total genes). Among the 110 DEG from cluster 3, 25 were also DE in at least one other cluster. Two dotplots show the 85 DEG specific to cluster 3 (left) or DE in one or more other B cell cluster (right) with an heatmap associating genes to the clusters in which they are DE. Dots are colorized based on the average expression of the gene in the cluster and dot size represents the percentage of cells expressing the gene. (B) Gene ontologies associated to downregulated genes in GZMB+ B cells from TOL B cells (cluster 3) using ClusterProfiler. Ontologies terms were reduced using rrvgo. The results are displayed using a heatmap of similarities between each significantly enriched GO (adjusted p.value < 0.05) coloured by similarities from blue (low similarity) to red (high similarity) No ontology was associated with upregulated genes.

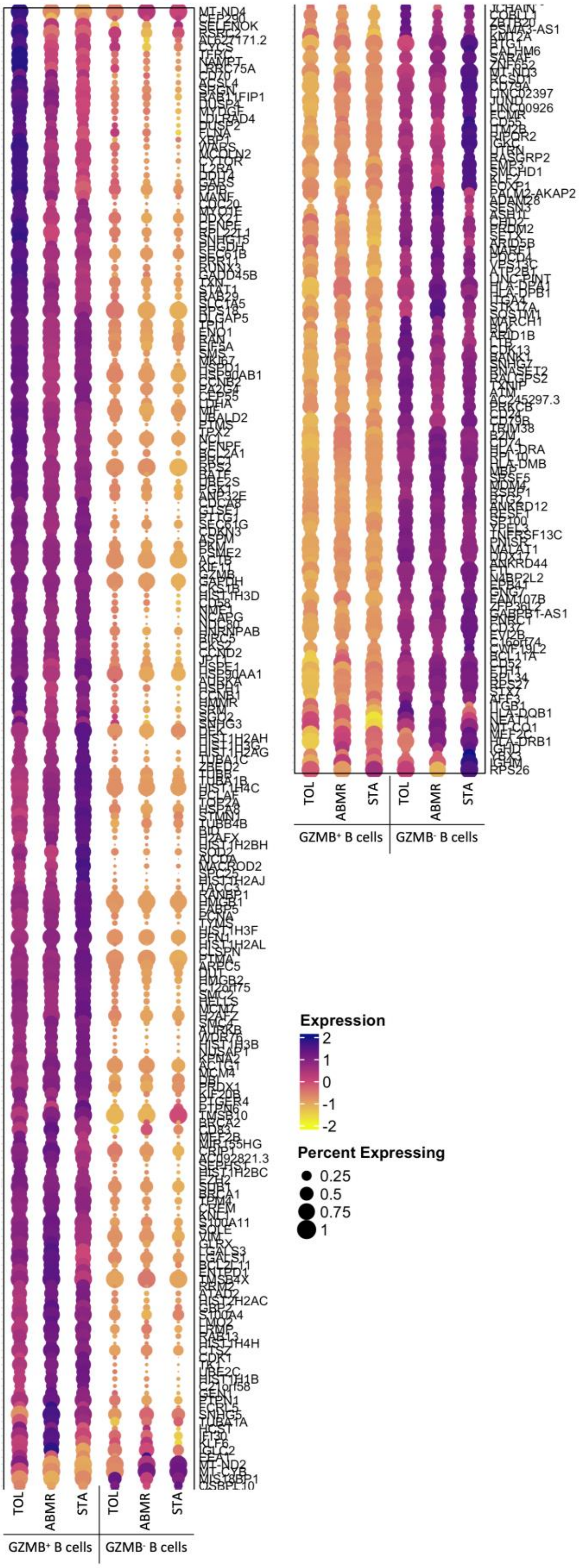

Figure S3: Dotplot showing differentially expressed genes between GZMB⁺ and GZMB⁻ B cells shared by all groups of transplanted patients
